# Identification of Vesicle Transport Proteins *via* Hypergraph Regularized K-Local Hyperplane Distance Nearest Neighbour Model

**DOI:** 10.3389/fgene.2022.960388

**Published:** 2022-07-13

**Authors:** Rui Fan, Bing Suo, Yijie Ding

**Affiliations:** ^1^ Institute of Fundamental and Frontier Sciences, University of Electronic Science and Technology of China, Chengdu, China; ^2^ Yangtze Delta Region Institute (Quzhou), University of Electronic Science and Technology of China, Quzhou, China; ^3^ Beidahuang Industry Group General Hospital, Harbin, China

**Keywords:** transport proteins, protein function prediction, hypergraph learning, local hyperplane, membrane proteins

## Abstract

The prediction of protein function is a common topic in the field of bioinformatics. In recent years, advances in machine learning have inspired a growing number of algorithms for predicting protein function. A large number of parameters and fairly complex neural networks are often used to improve the prediction performance, an approach that is time-consuming and costly. In this study, we leveraged traditional features and machine learning classifiers to boost the performance of vesicle transport protein identification and make the prediction process faster. We adopt the pseudo position-specific scoring matrix (PsePSSM) feature and our proposed new classifier hypergraph regularized k-local hyperplane distance nearest neighbour (HG-HKNN) to classify vesicular transport proteins. We address dataset imbalances with random undersampling. The results show that our strategy has an area under the receiver operating characteristic curve (AUC) of 0.870 and a Matthews correlation coefficient (MCC) of 0.53 on the benchmark dataset, outperforming all state-of-the-art methods on the same dataset, and other metrics of our model are also comparable to existing methods.

## 1 Introduction

Proteins are the basis of most life activities and perform important functions in different biochemical reactions. Proteins with different amino acid sequences and folding patterns have different functions. Understanding the factors that influence protein function has practical biological implications. Therefore, protein function prediction has been an important topic since the birth of bioinformatics. In recent years, machine learning-based protein function prediction methods have been widely used in many studies (Shen et al., 2019; Zhang J. et al., 2021; Zulfiqar et al., 2021; Ding et al., 2022b; Zhang et al., 2022), such as drug discovery (Ding et al., 2020c; Chen et al., 2021; Song et al., 2021; Xiong et al., 2021), protein gene ontology (Hong et al., 2020b; Zhang W. et al., 2021), DNA-binding proteins (Zou et al., 2021), enzyme proteins (Feehan et al., 2021; Jin et al., 2021), and protein subcellular localization (Ding et al., 2020b; Su et al., 2021; Wang et al., 2021; Zeng et al., 2022). In this study, we propose a novel method to identify vesicular transporters with machine learning.

Vesicular transport proteins are membrane proteins. The cell membrane separates the cell’s internal environment from the outside and controls the transport of substances into and out of the cell. Different substances enter and leave cells in different ways, and the transport of macromolecular substances is called vesicular transport. In vesicular transport, cells first surround substances and form vesicles. Vesicles move within cells and release their contents through vesicle rupture or membrane fusion. The process of vesicle transport exists widely in life activities. Vesicular transport proteins play an important role in vesicle transport by regulating the interactions of specific molecules with the vesicle membrane. In biology, there have been many studies on vesicular transport proteins, such as (Cheret et al., 2021; Li et al., 2021; Fu T. et al., 2022). Many human diseases are associated with abnormal vesicle transport proteins, such as those described in (Buck et al., 2021; Mazere et al., 2021; Zhou et al., 2022).

With the development of protein sequencing technology, an increasing number of vesicle transport protein sequences have been discovered. The need to rapidly identify vesicle transporter protein sequences conflicts with traditional experimental techniques, which are costly and time-consuming. Therefore, it is imperative to develop a fast and efficient computational method. To date, there have been few studies on the computational identification of vesicle transport proteins.

Computational identification of protein, RNA and DNA sequences has similar steps, and their processes can be described as two steps of feature extraction and classification. In 2019, Le et al. proposed a method (Vesicular-GRU) to identify vesicle transporters using position-specific scoring matrix (PSSM) features and a neural network classifier based on a convolutional neural network (CNN) and gated recurrent unit (GRU) and released the dataset used in their study (Le et al., 2019). In 2020, Tao et al. (Tao et al., 2020) attempted to classify vesicular transport proteins with fewer feature dimensions. Their model used the composition part of the method of composition, transition, and distribution (CTDC) features and a support vector machine (SVM) classifier. After dimensionality reduction with the Maximum Relevance Maximum Distance (MRMD) method, they obtained a comparatively satisfactory accuracy with fewer feature dimensions on the Le et al. dataset.

**FIGURE 1:**
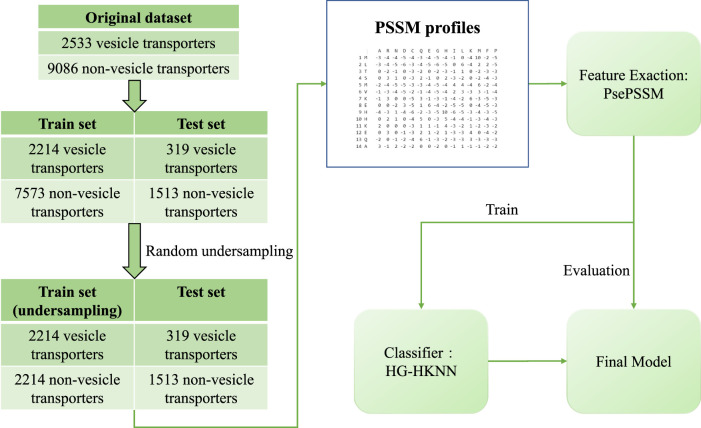
Flowchart of our model.

In our study, we propose a new model to identify vesicular transporters using pseudo position-specific scoring matrix (PsePSSM) features and a classifier called hypergraph regularized k-local hyperplane distance nearest neighbour (HG-HKNN). The main contributions of our work are as follows: 1) a better identification model of vesicle transport protein, with fewer feature dimensions and better results than the state-of-the-art model; and 2) a classifier called HG-HKNN that combines hypergraph learning (Zhou et al., 2006; Ding et al., 2020a) with k-local hyperplane distance nearest neighbours (HKNN) (Vincent and Bengio, 2001; Liu et al., 2021). The flowchart of our study is illustrated in Figure 1.

## 2 Materials and Methods

### 2.1 Dataset

The dataset we use to build and evaluate the model is the benchmark dataset released by Le et al. (Le et al., 2019). In the construction of the benchmark dataset, experimentally validated vesicular transport proteins were screened from the universal protein (UniProt) database (Consortium, 2019) and the gene ontology (GO) database (Consortium, 2004).

For the positive dataset, the authors collected protein sequences by searching the UniProt database for the keyword “vesicular transport” or the gene ontology term “vesicular transport”. Likewise, for the negative dataset, the authors collected a set of universal protein (membrane protein) sequences and excluded vesicular transporters from them. Next, protein sequences annotated by biological experiments were selected in the original dataset, and all protein sequences that were not validated experimentally were filtered out. The authors then eliminated homologous sequences on the positive and negative datasets, respectively, with a 30% cut-off level by the basic local alignment search tool (BLAST) clustering (Johnson et al., 2008). The BLAST clustering ensures that any two sequences in the dataset have less than 30% pairwise sequence similarity. Finally, protein sequences with noncanonical amino acids (X, U, B, Z) were removed from the dataset.

The benchmark dataset contains 2533 vesicular transport proteins and 9086 non-vesicular transport proteins, and the dataset is divided into a training set and a test set. The training set consists of 2144 vesicular transporters and 7573 non-vesicular transporters, and the test set consists of 319 vesicular transporters and 1513 non-vesicular transporters. We perform random undersampling (RUS) on the training set to balance the proportions of positive and negative samples. In random undersampling, we randomly select a sample from the class with more samples in the training set to represent its class, and repeat until there are the same number of vesicular transport proteins and non-vesicular transport proteins in the training set. The randomly undersampled training set has 2214 positive samples and 2214 negative samples. The details of the dataset are listed in Table 1.

**TABLE 1:** Details of the dataset used in our study.

	Original	Train Set	Train Set (RUS)	Test Set
Vesicular transport	2533	2214	2214	319
Non-vesicular transport	9086	7573	2214	1513

### 2.2 Feature Extraction

The feature type we use is PsePSSM (Chou and Shen, 2007), and the PSSM profile used to build PsePSSM is directly downloaded from the open-source data of Le et al. (Le et al., 2019). The authors of (Le et al., 2019) constructed these PSSM profiles by searching all sequences one by one in the non-redundant (NR) database with BLAST software. The PSSM matrix is an  L∗20 matrix similar to the following formula (Zhu et al., 2019). Each PSSM matrix corresponds to a protein sequence.

$$\begin{array}{c} P_{\mathrm{PSSM}}=\left[\begin{array}{cccc} E_{1\to 1} & E_{1\to 2} & \cdots & E_{1\to 20}\\ E_{2\to 1} & E_{2\to 2} & \cdots & E_{2\to 20}\\ \vdots & \vdots & \vdots & \vdots \\ E_{i\to 1} & E_{i\to 2} & \cdots & E_{i\to 20}\\ \vdots & \vdots & \vdots & \vdots \\ E_{L\to 1} & E_{L\to 2} & \cdots & E_{L\to 20} \end{array}\right]. \end{array}$$ (1)

In this formula, L is the length of the protein sequence. ${\text{E}}_{i\to j}$ represents the relationship between the amino acid at position i of the protein sequence and the amino acid of type j in the homologous sequence. j is the amino acid type number ranging from 1 to 20. The PSSM matrix contains the position-specific frequency information of amino acids in the protein homologous sequences, which is used to decode the evolutionary information of proteins. Compared with other protein information (such as amino acid frequency and physicochemical properties), the PSSM matrix of proteins not only contains the information of the proteins in the dataset but also contains the motif information of the protein homologous sequences in the NR database. However, the dimension of the PSSM matrix is too large, so further PsePSSM feature extraction is required.

The PsePSSM feature we use is a (ξ+1)*20 dimension feature, which can be calculated with this formula:

$$\begin{array}{c} P_{\mathrm{Pse}\mathrm{PSSM}}^{\xi }={\left[{\bar{E}}_{1}\cdots {\bar{E}}_{20}G_{1}^{1}\cdots G_{20}^{1}\cdots G_{1}^{\xi }\cdots G_{20}^{\xi }\right]}^{T}. \end{array}$$ (2)

where ${\bar{\text{E}}}_{j}$ is the average value of each column of the PSSM matrix, and the calculation of $G_{j}^{\xi }$ can be expressed by the following formula:

$$\begin{array}{c} G_{j}^{\xi }=\frac{1}{L-\xi }\overset{L-\xi }{\underset{i=1}{\sum }}{\left[E_{i\to j}-E_{\left(i+\xi \right)\to j}\right]}^{2}\;\left(j=1,2,\cdots ,20;\xi <L\right). \end{array}$$ (3)

$G_{j}^{\xi }$ is the correlation factor obtained by coupling the ξ th-most contiguous PSSM scores along the protein chain with amino acid type j . Clearly, ${\bar{\text{E}}}_{j}$ and $G_{j}^{0}$ are the same. Note that the maximum value of ξ must be less than the length of the shortest protein sequence in the benchmark dataset. The value of ξ we choose is 6, so $P_{PsePSSM}^{\xi }$ is a feature vector with 140 dimensions. When ξ increases, the evaluation metric first increases and then decreases and reaches the maximum value when ξ is 6.

### 2.3 Method for Classification

The hypergraph regularized k-local hyperplane distance nearest neighbour model (HG-HKNN) is a new classifier that combines the k-local hyperplane distance nearest neighbour algorithm (HKNN) and hypergraph learning.

#### 2.3.1 HKNN

**FIGURE 2:**
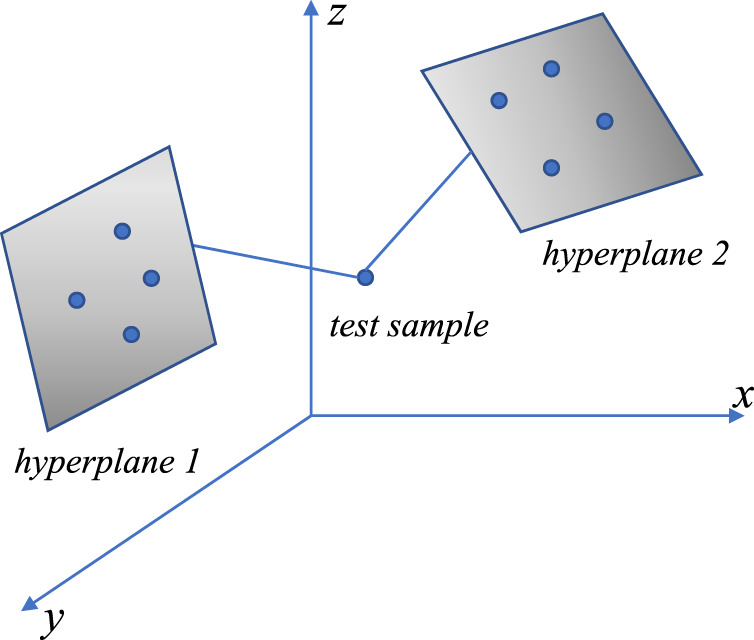
Sketch of an HKNN.

In the HKNN (Vincent and Bengio, 2001) workflow, multiple hyperplanes are constructed first, each hyperplane corresponds to a class in the training set, and the hyperplane is constructed by the k samples of the same class that is closest to the test sample. Then, the HKNN predicts the class of the test sample by comparing the distance between the test sample and the hyperplanes and assigns the test sample to the class corresponding to the nearest hyperplane (Ding et al., 2022c). Figure 2 shows a sketch of an HKNN, where sample x obtains its class by comparing the distances to hyperplane 1 and hyperplane 2.

In class c , when x represents the test sample, the hyperplane can be expressed as the following formula:

$$\begin{array}{c} LH_{k}^{c}\left(x\right)=\left\{p^{c}|p^{c}={\bar{N}}^{c}+\overset{k}{\underset{i=1}{\sum }}\alpha_{i}^{c}V_{i}^{c},\alpha_{1\ldots k}^{c}\in R^{k}\right\}. \end{array}$$ (4)

where k means that k nearest neighbour samples are taken to construct the hyperplane, and the i -th sample in class c can be expressed as $N_{i}^{c}$ ( i from 1 to k ). Let ${\bar{N}}^{c}$ represent the centre of $N_{i}^{c}$ , and let $V_{i}^{c}=N_{i}^{c}-{\bar{N}}^{c}$ , where $\alpha_{i}^{c}$ is an undetermined parameter; then, $p^{c}$ is a point on this hyperplane.

The mean squared distance of the test sample x to each hyperplane can be expressed as follows:

$$\begin{array}{c} {\left(LH_{k}^{c}\left(x\right)\right)}^{2}={\left\|x-{\bar{N}}^{c}-\overset{k}{\underset{i=1}{\sum }}\alpha_{i}^{c}V_{i}^{c}\right\|}^{2}+\lambda \overset{k}{\underset{i=1}{\sum }}{\left(\alpha_{i}^{c}\right)}^{2}. \end{array}$$ (5)

where λ is the regularization parameter of $\alpha_{i}^{c}$ , which is used to reduce the complexity of the model. $\alpha^{c}$ is obtained by minimizing the distance. Finally, the classification result of the HKNN can be judged by the following formula:

$$\begin{array}{c} c=argmin_{c}{\left\|x-{\bar{N}}^{c}-\overset{k}{\underset{i=1}{\sum }}\alpha_{i}^{c}V_{i}^{c}\right\|}^{2}. \end{array}$$ (6)

HKNN has relatively good performance on unbalanced datasets because the same number of samples are selected in each class. However, since the distribution of samples cannot be fully expressed by a hyperplane, the performance of the HKNN is disturbed by the distribution of samples.

#### 2.3.2 Hypergraph Learning

In machine learning, we can express the similarity between two samples by calculating the inner product of the features of the two samples to form a pairwise similarity matrix (Yang et al., 2020). However, the relationship between samples cannot simply be determined by pairwise similarity. Therefore, hypergraphs (Zhou et al., 2006) are proposed to express the relationship between three or more samples.

**FIGURE 3:**
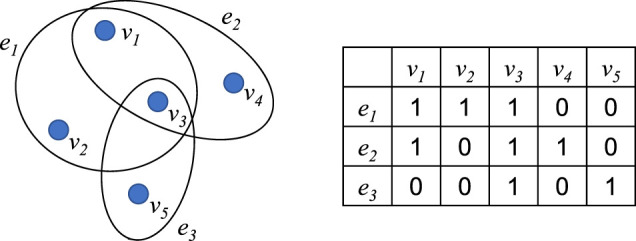
A hypergraph and its association matrix H.

In a hypergraph, each hyperedge consists of multiple vertices. Figure 3 is a hypergraph and its association matrix H . In our study, each hyperedge weights 1. When hyperedge $e_{j}$ contains vertex $v_{i}$ , then $H_{ij}$ is 1; otherwise, it is 0.

Formally, the association matrix H , the degree of each hyperedge, and the degree of each vertex can be expressed as:

$$\begin{array}{c} H\left(v,e\right)=\{\begin{array}{cc} 1, & \;\mathrm{if}\;v\in e\\ 0, & \;\mathrm{if}\;v\notin e \end{array}, \end{array}$$ (7a)

$$\begin{array}{c} \delta \left(e\right)=\underset{v\in V}{\sum }H\left(v,e\right), \end{array}$$ (7b)

$$\begin{array}{c} d\left(v\right)=\underset{e\in E}{\sum }H\left(v,e\right). \end{array}$$ (7c)

The Laplacian matrix of a hypergraph association matrix H can be calculated as:

$$\begin{array}{c} L_{H}=I-D_{v}^{-\frac{1}{2}}HAD_{e}^{-1}H^{T}D_{v}^{-\frac{1}{2}}. \end{array}$$ (8)

where $D_{v}$ and $D_{e}$ are the diagonal matrices formed by d(v) and δ(e) , respectively, and A is the same as the identity matrix I in our study. We construct the association matrix H with the k -nearest neighbour algorithm proposed by Zhou et al. (Zhou et al., 2006). Given a set of samples, we choose the k nearest neighbours of each sample and construct a hyperedge containing these k vertices. Finally, we construct N hyperedges for a dataset of N samples.

#### 2.3.3 HG-HKNN

The HG-HKNN rewrites the mean squared distance from the test sample x to each hyperplane in the HKNN into the following form:

$$\begin{array}{c} {\left(LH_{k}^{c}\left(x\right)\right)}^{2}={\left\|\phi \left(\overset{-}{x}\right)-\overset{k}{\underset{i=1}{\sum }}\alpha_{i}^{c}\phi \left(V_{i}^{c}\right)\right\|}^{2}+\lambda \overset{k}{\underset{i=1}{\sum }}{\left(\alpha_{i}^{c}\right)}^{2}+\mu \overset{k}{\underset{p=1}{\sum }}\overset{k}{\underset{q=1}{\sum }}w_{p,q}^{c}{\left(\alpha_{p}^{c}-\alpha_{q}^{c}\right)}^{2}. \end{array}$$ (9)

The kernel trick (Hofmann, 2006; Ding et al., 2019) is used to solve this problem, and the map ϕ maps the feature space to higher dimensions. $\overset{-}{x}=x-{\overset{-}{N}}^{c}$ is a simple rewrite. The third term in this formula is the Laplacian regularization term, which improves classification performance by smoothing the feature space (Ding et al., 2021). μ is the Laplacian regularization parameter, and $w_{p,q}^{c}$ is the similarity between the p -th nearest and the q -th nearest samples in the k samples in class c , which is calculated by the kernel function (Ding et al., 2022a). K(x,y)=ϕ(x),ϕ(y) represents the kernel function, which is the radial basis function (RBF) in our study.

By minimizing the distance and making the partial derivative of ${\left(LH_{k}^{c}\left(x\right)\right)}^{2}$ with respect to $\alpha^{c}$ zero, then the solution of $\alpha^{c}$ is obtained as follows:

$$\begin{array}{c} \frac{\partial \left({\left(LH_{k}^{c}\left(x\right)\right)}^{2}\right)}{\partial \alpha^{c}}=0,\\ \left(\phi {\left(V^{c}\right)}^{T}\phi \left(V^{c}\right)+\lambda I+\mu L\right)\alpha^{c}=\phi {\left(V^{c}\right)}^{T}\phi \left(\overset{-}{x}\right),\\ \alpha^{c}={\left(\phi {\left(V^{c}\right)}^{T}\phi \left(V^{c}\right)+\lambda I+\mu L\right)}^{-1}\phi {\left(V^{c}\right)}^{T}\phi \left(\overset{-}{x}\right),\\ \begin{array}{c} \alpha^{c}={\left(K\left(V^{c},V^{c}\right)+\lambda I+\mu L\right)}^{-1}K\left(V^{c},\overset{-}{x}\right). \end{array} \end{array}$$ (10)

We construct the hypergraph and use the Laplacian matrix of the hypergraph to replace the Laplacian matrix in the above formula:

$$\begin{array}{c} \alpha^{c}={\left(K\left(V^{c},V^{c}\right)+\lambda I+\mu L_{H}\right)}^{-1}K\left(V^{c},\overset{-}{x}\right). \end{array}$$ (11)

Note that the original Laplacian matrix contains pairwise similarities between samples, while our hypergraph Laplacian matrix contains more complex relationships between samples.

Now the distance from sample x to the c -th hyperplane can be expressed as follows:

$$\begin{array}{c} distance_{c}=\left\|\phi \left(\tilde{x}\right)-\overset{k}{\underset{i=1}{\sum }}\alpha_{i}^{c}\phi {\left(V_{i}^{c}\right)}^{2}\right\|,\\ ={\left(\phi \left(\bar{x}\right)-\phi \left(V^{c}\right)\alpha^{c}\right)}^{T}\left(\phi \left(\bar{x}\right)-\phi \left(V^{c}\right)\alpha^{c}\right),\\ =\left(K\left(\overset{-}{x},\overset{-}{x}\right)-2{\left(\alpha^{c}\right)}^{T}K\left(V^{c},\overset{-}{x}\right)+{\left(\alpha^{c}\right)}^{T}K\left(V^{c},V^{c}\right)\alpha^{c}\right). \end{array}$$ (12)

Finally, we assign the test sample x to class c :

$$\begin{array}{c} c=argmin_{c}\left(distance_{c}\right). \end{array}$$ (13)

We define the prediction score as follows:

$$\begin{array}{c} score_{c}=\frac{\sqrt{distance_{c}}}{\sum_{i=1}^{C}\sqrt{distance_{i}}},i=1,\;2,\;\ldots ,\;C. \end{array}$$ (14)

The process of HG-HKNN is listed in Algorithm 1

Algorithm 1 Algorithm of HG-HKNN

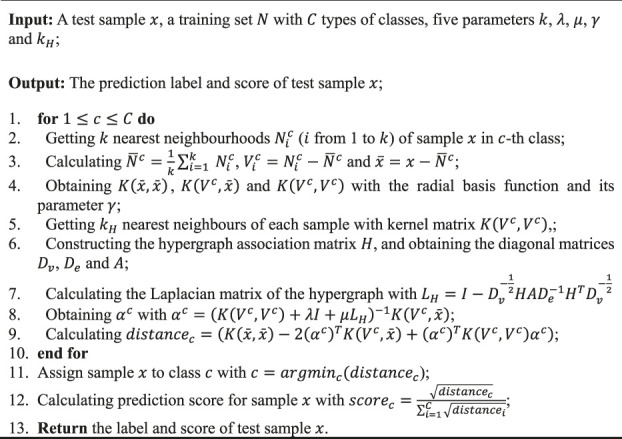

## 3 Results and Discussion

### 3.1 Evaluation

In this section, we will introduce the evaluation methods and metrics we use. We use positive to describe vesicular transport proteins and negative to describe non-vesicular transport proteins. We optimize the parameters with cross-validation (CV) on the training set and then evaluate our model on the test set.

Cross-validation sets aside a small portion of the dataset for validating the model, while the rest of the dataset is used for training the model (Zhang D. et al., 2021; Lv et al., 2021; Yang et al., 2021; Zheng et al., 2021; Li F. et al., 2022; Li X. et al., 2022). The leave-one-out cross-validation (LOOCV) is a classic cross-validation method (Qiu et al., 2021). LOOCV takes only one sample in the dataset at a time for validation and uses other samples in the dataset to train the model. Until all samples are left out once for validation, the leave-one-out method obtains statistical values for multiple results. However, the leave-one-out method is too time-consuming, so we adopted another cross-validation method: k -fold cross-validation (K-CV). K-CV divides the dataset into k subsets. Each time, one of the subsets is taken for validation, and the remaining k−1 subsets are used for training the model. In this way, k prediction results are obtained, and we take the average of these k results as the result of k -fold cross-validation.

The evaluation indicators we take include sensitivity, precision, specificity, accuracy (ACC), Matthews correlation coefficient (MCC), and area under the receiver operating characteristic curve (AUC), which have been widely used in previous studies (Hong et al., 2020a; Tang et al., 2020; Pan et al., 2022; Song et al., 2022).

$$\begin{array}{c} sensitivity=\frac{TP}{TP+FN}, \end{array}$$ (15a)

$$\begin{array}{c} precision=\frac{TP}{TP+FP}, \end{array}$$ (15b)

$$\begin{array}{c} specificity=\frac{TN}{TN+FP}, \end{array}$$ (15c)

$$\begin{array}{c} ACC=\frac{TP+TN}{TP+FN+FP+FN}, \end{array}$$ (15d)

$$\begin{array}{c} MCC=\frac{1-\left(\left(\frac{FN}{TP}+FN\right)+\left(\frac{FP}{TN}+FP\right)\right)}{\sqrt{\left(1+\left(FP-\frac{FN}{TP}+FN\right)\right)\left(1+\left(FN-\frac{FP}{TN}+FP\right)\right)}}. \end{array}$$ (15e)

where TP, FP, TN, and FN represent true positives, false positives, true negatives, and false negatives, respectively. In addition, the AUC is obtained by integrating the receiver operating characteristic curve (ROC) (Fu J. et al., 2022). The ROC curve plots sensitivity and specificity at different classification thresholds (Tzeng et al., 2022). The more meaningful ones are AUC and precision since our test set is a class-imbalanced dataset. In our model, we perform 10-fold cross-validation on a training set of 4428 samples (2214 positive and 2214 negative). The binary classification threshold is set to the default 0.5. Finally, the trained model is evaluated on the test set, which has 319 positive samples and 1513 negative samples.

### 3.2 Parameter Tuning

In this section, we describe the parameter tuning process for our model. Classification metrics are largely influenced by parameter tuning. The HG-HKNN has five parameters: k , λ , μ , γ , and $k_{H}$ . k represents the number of neighbour samples selected when constructing the hyperplane. λ is the regularization parameter in $L_{2}$ regularization and μ is the Laplacian regularization parameter. γ is a parameter in the radial basis function. $k_{H}$ is the number of neighbours used to construct the hypergraph.

We first adjust the k parameters among them. We set k , μ and γ to be 0.2, 0.2 and 0.2, respectively, and $k_{H}$ to be 2. We perform 10-fold cross-validation for different values of k , and the best parameter k is determined to be 650; the details are shown in Table 2.

**TABLE 2:** Details in parameter tuning of k .

k	AUC	ACC	Precision	Specificity
200	0.8127	0.7256	0.7677	0.8035
350	0.8241	0.7319	0.7897	0.8311
500	0.8284	0.7362	0.7940	0.8338
650	0.8292	0.7398	0.7954	0.8333
800	0.8287	0.7425	0.7927	0.8265
950	0.8279	0.7437	0.7840	0.8134

For λ , μ , γ and $k_{H}$ , we adopt the grid search method for parameter tuning. The grid search method enumerates the possible values of each parameter, combines the possible values of all parameters into groups, and then trains the model with each group of parameters to obtain the best set of parameters. In our grid search, the possible values of λ , μ and γ are all 0.1, 0.2, 0.4, and 0.8, and the $k_{H}$ values in the hypergraph range from 2 to 10. The best parameters for choosing λ , μ and γ are 0.4, 0.4 and 0.4, respectively. The best parameter $k_{H}$ is 2, and the best AUC is 0.8309.

In our dataset, the dimension of features is much smaller than the number of samples, which is regarded as a sign that the dataset is linearly inseparable. On linearly inseparable datasets, the RBF kernel generally performs better than the linear or polynomial kernel. Formally, the Laplacian kernel is similar to the RBF kernel, and they usually have similar performance, but the Laplacian kernel function requires additional computational cost. We regard the type of kernel function used by HG-HKNN as an additional hyperparameter and conduct comparative experiments. The details of the experimental results are shown in Table 3. The results show that the RBF kernel has the best performance.

**TABLE 3:** Comparison of classification metrics among different kernels.

Kernel Type	AUC	MCC	ACC	Precision	Specificity
Linear	0.7618	0.3739	0.6719	0.7833	0.8686
Polynomial	0.8021	0.4664	0.7322	0.7519	0.7687
Laplacian	0.8243	0.5153	0.7575	0.7592	0.7597
RBF	0.8309	0.5099	0.7538	0.7760	0.7922

### 3.3 Comparison With Traditional Machine Learning Methods

In the previous section, we have chosen the best parameters for our model. Our model is trained with traditional PsePSSM features, with nothing special in feature extraction. In this section, to highlight the effect of our proposed classifier HG-HKNN, we train some models with different traditional machine learning classifiers, the same training set, and the same PsePSSM feature extraction method. We perform 10-fold cross-validation on these models and compare the evaluation metrics of these models with ours. Note that the only difference between these models is the classifier.

We implement and train these models with the programming language’s built-in library of functions. With the help of the parameter optimization function, we can automatically train the SVM model with the best evaluation metrics. After parameter tuning, the parameters in the other models are as follows: K=20 in the k-nearest neighbour model(KNN), ntrees=60 in the random forest model (RF), and k=30 and λ=10 in HKNN. Table 4 shows the comparison of our model with other traditional machine learning models in 10-fold cross-validation.

**TABLE 4:** Comparison of classification metrics among different models.

Techniques	AUC	MCC	ACC	Precision	Specificity
KNN	0.7824	0.4189	0.7078	0.6886	0.6519
RF	0.8019	0.4576	0.7285	0.7267	0.7231
SVM	0.8091	0.4820	0.7405	0.7466	0.7502
HKNN	0.8203	0.4976	0.7484	0.7442	0.7371
OG-HKNN	0.8289	0.4944	0.7446	0.7843	0.8130
HG-HKNN	0.8309	0.5099	0.7538	0.7760	0.7922

Among them, the prediction effect of HKNN is better than that of the KNN algorithm. Intuitively explained in principle, although the classical K-nearest neighbour algorithm can fit the training samples well, it does not work well for the unseen samples located near the decision boundary. This is the overfitting problem of the KNN algorithm, and overfitting is more obvious in small data sets. HKNN constructs a hyperplane for k-nearest neighbour samples and then compares the distances between the test sample and the hyperplanes. The construction of the hyperplane can be analogous to adding more sample points to the k-nearest neighbours, which will reduce the interference of extreme samples on the decision boundary. Therefore, compared with KNN, the HKNN model has a smoother decision boundary, avoiding the disadvantage of overfitting in KNN.

Our proposed HG-HKNN model outperforms the other models on almost all metrics at the same level of comparison. By introducing Laplacian regularization in manifold learning, the HG-HKNN model incorporates local similarity information in the feature space into the construction process of the hyperplane. Compared with the HKNN model, the HG-HKNN model not only reduces the disturbance of extreme samples to the decision boundary, but also preserves the local similarity information in the feature space. In the HG-HKNN model, we replace the ordinary graph with a hypergraph for Laplacian regularization. Hypergraph learning allows us to represent feature space local structures with more complex relationships than just pairwise similarity relationships. This further improves the performance of our HG-HKNN model. To highlight the effect of hypergraph learning, we add an ordinary graph regularized HKNN model (OG-HKNN) to our comparison, and the details are also listed in Table 4. The parameter tuning process of the OG-HKNN model is the same as that of the HG-HKNN. The best parameters for choosing λ , μ , γ and k are 0.2, 0.8, 0.4 and 350, respectively. The experimental results show that the AUC, MCC and ACC of the HG-HKNN model are better than the OG-HKNN model.

One disadvantage of our model is that HG-HKNN increases computation time and memory usage compared to HKNN. In terms of memory usage, the storage of hypergraphs, Laplacian matrices, and kernel matrices in HG-HKNN increases memory usage. In terms of operating efficiency, we conduct experiments on the test set with the same parameter k=20 , HKNN completes the computation in 362 milliseconds, while HG-HKNN completes the computation in 640 milliseconds. Such computational time cost is acceptable, especially considering the performance of HG-HKNN and time-consuming deep learning models in vesicle transporter identification.

### 3.4 Comparison With Previous Techniques

In this section, we aim to compare our model with previous techniques to highlight the performance of our proposed model on benchmark datasets. After optimizing the parameters with cross-validation, we obtain the optimal values of each parameter in HG-HKNN, where λ is 0.4, k is 650, γ is 0.4, μ is 0.4, and the value of $k_{H}$ in the hypergraph part is 2. With these parameters, we no longer perform cross-validation on the training set but instead feed the entire training set into our model and then evaluate our final model on the test set. Among the metrics, the AUC is 87.0%, and the MCC is 0.53. Compared with the existing state-of-the-art Vesicular-GRU method with an AUC of 86.1% and MCC of 0.52, our model has higher AUC and MCC values, fewer feature dimensions (140 dimensions) and fewer parameters.

We compare our model with several other existing methods, among which the GRU model is a prediction method using traditional PSSM features and GRU and BLAST is a general-purpose protein prediction tool (Johnson et al., 2008). BLSTM is a commonly used prediction method in protein research (Li et al., 2020). The state-of-the-art method Vesicular-GRU (Le et al., 2019), a prediction method based on 1D CNN and GRU, is also listed in the comparison. The details of the comparison are shown in Table 5.

**TABLE 5:** Comparison of our model with other existing technologies.

Techniques	AUC	MCC	ACC	Sensitivity	Precision	Specificity
GRU	0.848	0.44	79.2	70.8	44.0	81.0
BLSTM	0.846	0.46	84.6	54.2	55.8	90.9
BLAST	0.82	0.43	83.6	54.1	52.8	89.8
Vesicular-GRU	0.861	0.52	82.3	79.2	48.7	82.9
HG-HKNN	0.870	0.53	84.1	72.1	53.2	86.7

The meaning of the indicators has been described in the previous section. Experimental results show that our model achieves the best AUC and MCC metrics on this imbalanced benchmark dataset. Deep learning is involved in most of the methods in the comparison. The black box is an unavoidable problem for deep learning-based methods, and it is difficult to intuitively understand which factors lead to the predicted results. In deep learning models, researchers need to optimize a large number of parameters to improve the performance of the network, and these parameters are directly tuned through back-propagation of the prediction results, resulting in overfitting and the curse of dimensionality. The neural network in the Vesicular-GRU model has hundreds of thousands of parameters, which makes the Vesicular-GRU model a potential risk of overfitting on the training set. Our HG-HKNN has only five parameters, and the performance of our model is mainly attributable to hypergraph regularization and hyperplane rather than fitting to the parameters. Local hyperplane models have better performance on imbalanced datasets because the same number of samples are selected in each class. Like many biological sequence datasets, the vesicle transporter dataset is a typically imbalanced dataset, which is where the local hyperplane model excels. Furthermore, HG-HKNN applies kernel tricks to handle high-dimensional features, avoiding the curse of dimensionality. Although there is an increase in time and memory usage compared to HKNN, our model is faster relative to deep learning models trained with huge parameters via backpropagation. With only five parameters, our model avoids the black box, overfitting and curse of dimensionality problems in deep learning and makes predictions faster, and the performance of our model is equal to or higher than all the mentioned techniques, especially in terms of MCC and AUC.

## 4 Conclusion

In this study, we propose a novel approach for predicting vesicular transport proteins. The existing methods are typically performed with complex neural networks or by extracting a large number of features. Our method classifies vesicular transport proteins with PsePSSM features and our proposed HG-HKNN model. We completed the prediction of vesicle transporters with only 140-dimensional features and 5 parameters with satisfactory results. Experimental results show that our method has the best AUC of 0.870 and MCC of 0.53 on the benchmark dataset and outperforms the state-of-the-art method (Vesicular-GRU) in ACC, MCC and AUC. Other metrics of our model are also comparable to other methods. A traditional machine learning computational model is used in our approach, avoiding some of the drawbacks of deep learning. Compared with another study (Tao et al., 2020) using traditional machine learning on the same dataset, their study achieved 72.2% accuracy and 0.34 MCC with 21-dimensional CTDC features after MRMD (He et al., 2020) dimensionality reduction, while our model achieves 84.1% accuracy and 0.53 MCC with 140-dimensional PsePSSM features. Furthermore, like CTDC features, the classical features we used imply that amino acids have a certain regularity in the arrangement of the protein sequence. Since PSSM matrix information is a commonly used motif representation, our study may help scholars to judge whether an unknown protein is a vesicle transporter.

The proposed method also has the following limitations: 1) In the case of large parameter k, the prediction takes a long time; 2) Our model uses the PsePSSM feature without incorporating sequence information for prediction; and 3) Feature selection and dimensionality reduction are not performed in our model. For the first limitation, parallel optimization can be used to solve the problem of computation time. For the second question, adding sequence features such as amino acid frequency or composition of k-spaced amino acid pairs (CKSAAP) to our model may further improve the prediction accuracy. For the third question, the dataset can be processed with feature selection and dimensionality reduction tools that remove redundant features. The results of this study can provide a basis for further studies in computational biology to identify vesicle transport proteins with classical features and traditional machine learning classifiers.

## Data Availability

Our experimental code can be obtained from https://github.com/ferryvan/HG-HKNN, and the datasets used in this study can be found in (Le et al., 2019).
