# Supplementary material for: Identification of Vesicle Transport Proteins via Hypergraph Regularized K-Local Hyperplane Distance Nearest Neighbour Model
Source: Front Genet. 2022 Jul 13;13:960388. doi: 10.3389/fgene.2022.960388 (PMC9326258; doi:10.3389/fgene.2022.960388)
Supplement: Supplementary file 1 [file Presentation1.PPTX]

## Slide 1
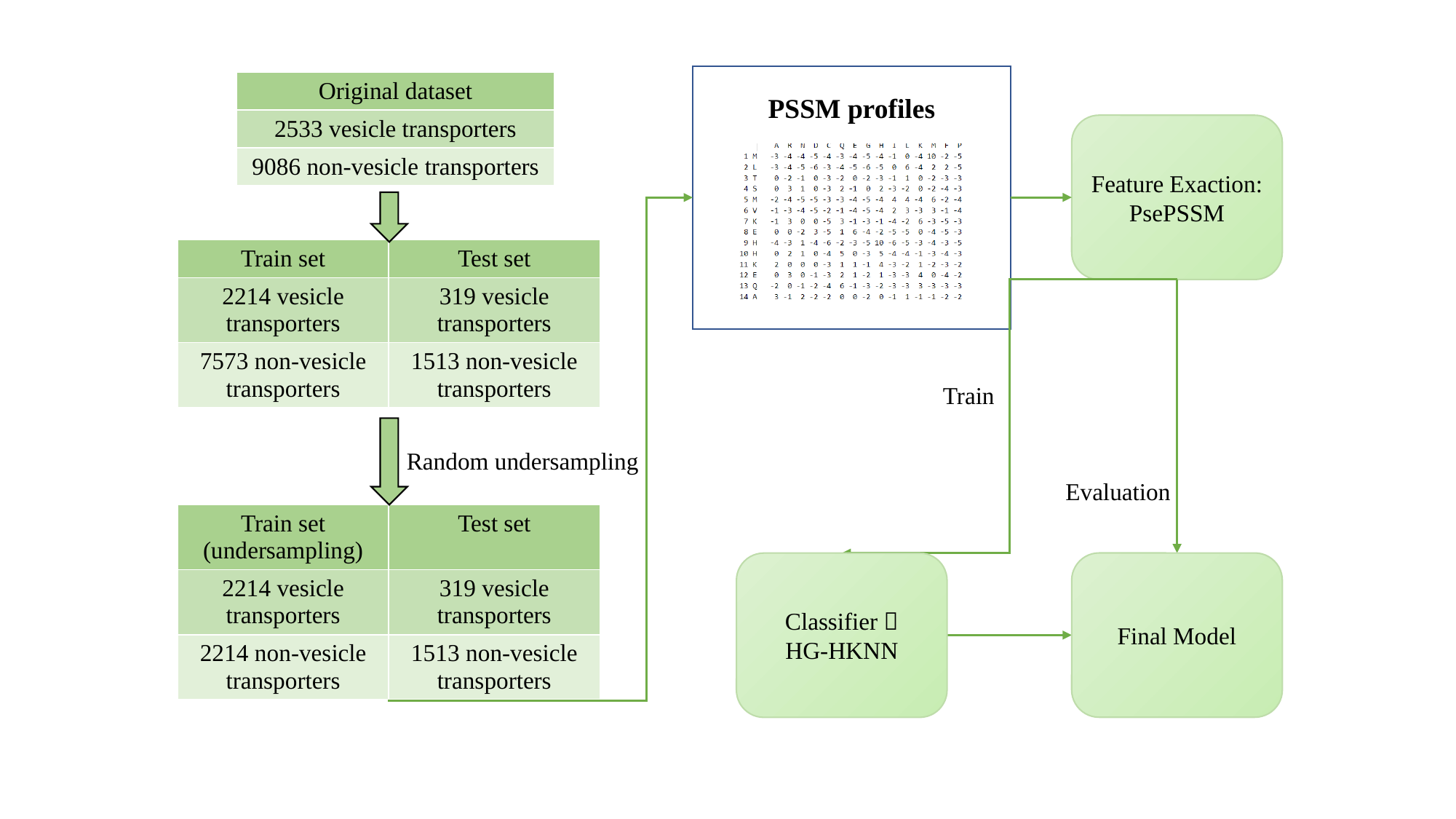

| Original dataset |
| --- |
| 2533 vesicle transporters |
| 9086 non-vesicle transporters |
PSSM profiles
Feature Exaction:
PsePSSM
| Train set | Test set |
| --- | --- |
| 2214 vesicle transporters | 319 vesicle transporters |
| 7573 non-vesicle transporters | 1513 non-vesicle transporters |
Train
Random undersampling
Evaluation
| Train set (undersampling) | Test set |
| --- | --- |
| 2214 vesicle transporters | 319 vesicle transporters |
| 2214 non-vesicle transporters | 1513 non-vesicle transporters |
Final Model
Classifier：
HG-HKNN
