# Supplementary figures and images for: Identification of Vesicle Transport Proteins via Hypergraph Regularized K-Local Hyperplane Distance Nearest Neighbour Model
Source: Front Genet. 2022 Jul 13;13:960388. doi: 10.3389/fgene.2022.960388 (PMC9326258; doi:10.3389/fgene.2022.960388)

## Slide 1
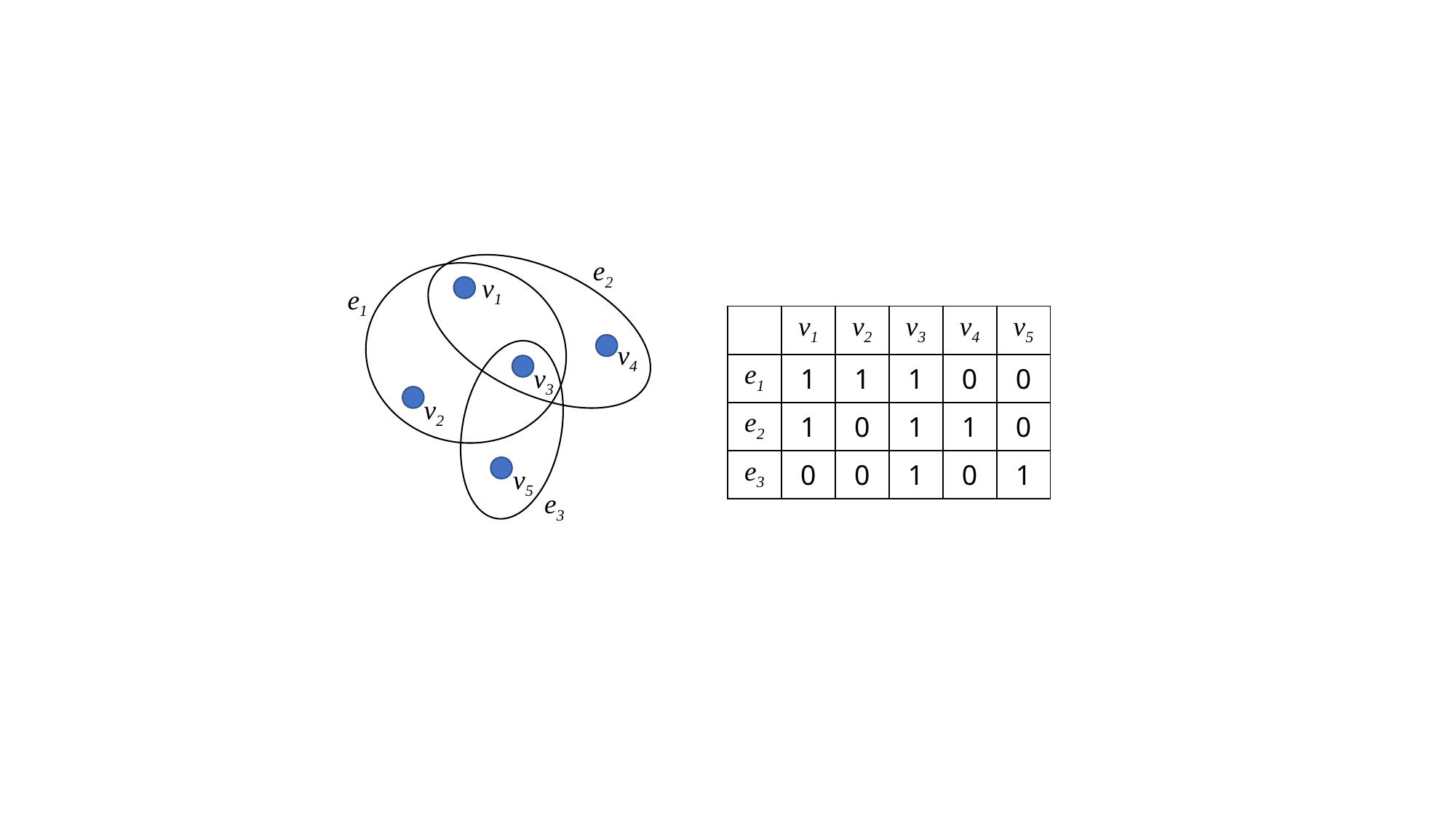

e2
v1
e1
| | v1 | v2 | v3 | v4 | v5 |
| --- | --- | --- | --- | --- | --- |
| e1 | 1 | 1 | 1 | 0 | 0 |
| e2 | 1 | 0 | 1 | 1 | 0 |
| e3 | 0 | 0 | 1 | 0 | 1 |
v4
v3
v2
v5
e3

Supplement: Supplementary file 2 [file Presentation3.PPTX]

## Slide 1
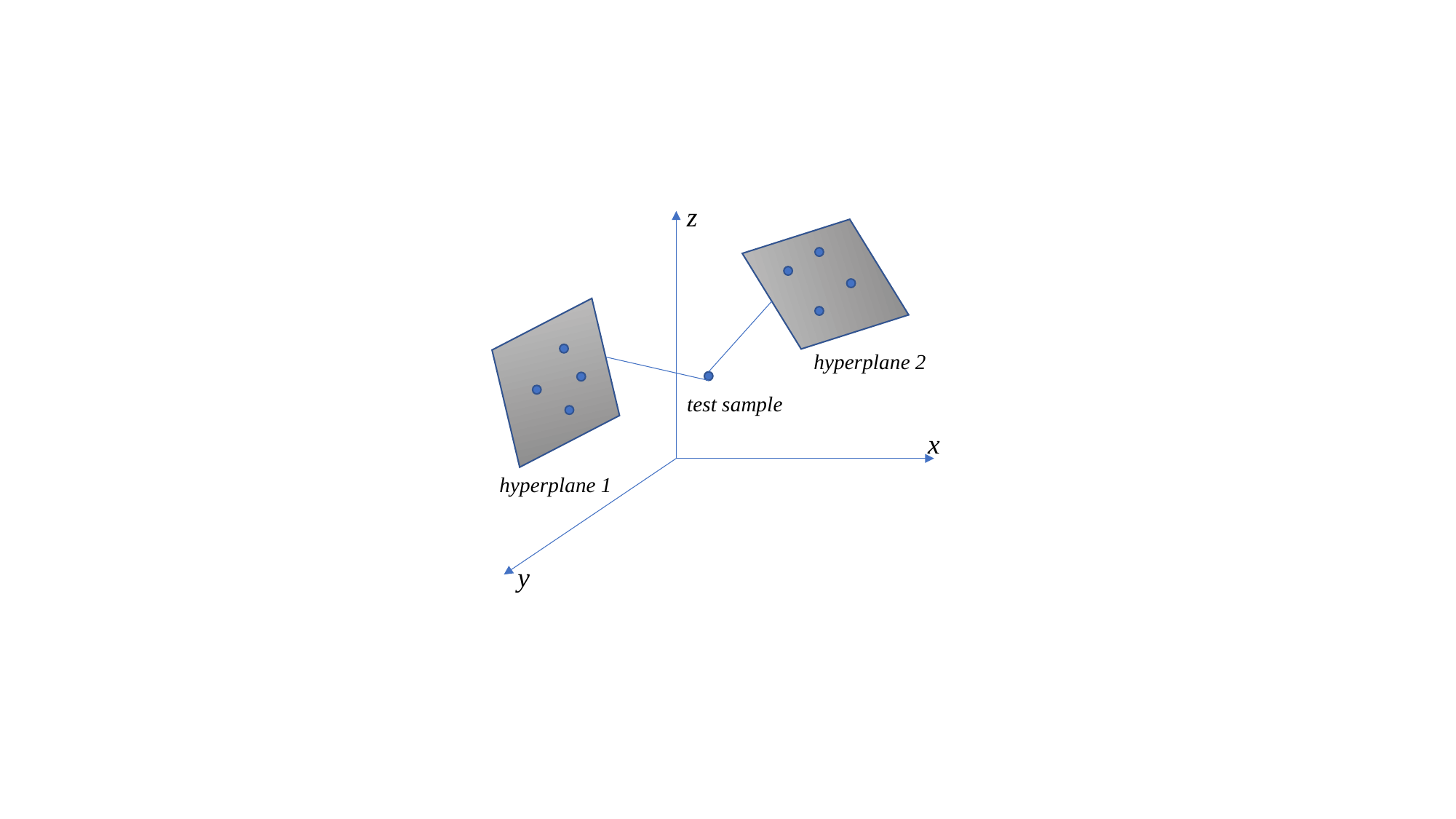

z
hyperplane 2
test sample
x
hyperplane 1
y

Supplement: Supplementary file 3 [file Presentation2.PPTX]
